# Natural Compounds and Products from an Anti-Aging Perspective

**DOI:** 10.3390/molecules27207084

**Published:** 2022-10-20

**Authors:** Geir Bjørklund, Mariia Shanaida, Roman Lysiuk, Monica Butnariu, Massimiliano Peana, Ioan Sarac, Oksana Strus, Kateryna Smetanina, Salvatore Chirumbolo

**Affiliations:** 1 Council for Nutritional and Environmental Medicine (CONEM), Toften 24, 8610 Mo i Rana, Norway; 2 Department of Pharmacognosy and Medical Botany, I. Horbachevsky Ternopil National Medical University, 46001 Ternopil, Ukraine; 3 Department of Pharmacognosy and Botany, Danylo Halytsky Lviv National Medical University, 79010 Lviv, Ukraine; 4 CONEM Ukraine Life Science Research Group, Danylo Halytsky Lviv National Medical University, 79010 Lviv, Ukraine; 5 Chemistry & Biochemistry Discipline, Banat’s University of Agricultural Sciences and Veterinary Medicine “King Michael I of Romania” from Timisoara, 300645 Timisoara, Romania; 6 CONEM Romania Biotechnology and Environmental Sciences Group, University of Life Sciences “King Mihai I” from Timisoara, 300645 Timisoara, Romania; 7 Department of Chemical, Physical, Mathematical and Natural Sciences, University of Sassari, 07100 Sassari, Italy; 8 Department of Drug Technology and Biopharmaceutics, Danylo Halytsky Lviv National Medical University, 79010 Lviv, Ukraine; 9 Department of Organic Chemistry and Pharmacy, Lesya Ukrainka Volyn National University, 43025 Lutsk, Ukraine; 10 Department of Neurosciences, Biomedicine and Movement Sciences, University of Verona, 37134 Verona, Italy; 11 CONEM Scientific Secretary, Strada Le Grazie 9, 37134 Verona, Italy

**Keywords:** aging, antioxidants, cosmetic ingredients, medicinal plants, probiotics, natural compounds, oxidative stress

## Abstract

Aging is a very complex process that is accompanied by a degenerative impairment in many of the major functions of the human body over time. This inevitable process is influenced by hereditary factors, lifestyle, and environmental influences such as xenobiotic pollution, infectious agents, UV radiation, diet-borne toxins, and so on. Many external and internal signs and symptoms are related with the aging process and senescence, including skin dryness and wrinkles, atherosclerosis, diabetes, neurodegenerative disorders, cancer, etc. Oxidative stress, a consequence of the imbalance between pro- and antioxidants, is one of the main provoking factors causing aging-related damages and concerns, due to the generation of highly reactive byproducts such as reactive oxygen and nitrogen species during the metabolism, which result in cellular damage and apoptosis. Antioxidants can prevent these processes and extend healthy longevity due to the ability to inhibit the formation of free radicals or interrupt their propagation, thereby lowering the level of oxidative stress. This review focuses on supporting the antioxidant system of the organism by balancing the diet through the consumption of the necessary amount of natural ingredients, including vitamins, minerals, polyunsaturated fatty acids (PUFA), essential amino acids, probiotics, plants’ fibers, nutritional supplements, polyphenols, some phytoextracts, and drinking water.

## 1. Introduction

Aging is a natural, evolutionarily programmed phenomenon, leading to a senescent phenotype, characterized by involutive events such as tissue degeneration, telomers’ shortening, dementia and cognitive deficits, functional impairments, and chronic pathologies [1,2]. Aging is therefore a degenerative process, which was particularly investigated in recent years, and for which numerous theories have been formulated concerning its programmed or non-programmed character.

Theories of programmed aging are subdivided into three conceptual sub-categories: (a) a theory regarding a genetically programmed longevity which assumes that aging is the consequence of starting or stopping certain genes, including the role of genetic instability (shortening of telomeres) in the dynamics of aging processes; (b) an endocrine theory—according to which, aging is governed by a biological clock whose function is regulated by endocrine mechanisms, among which the insulin-like growth hormone IGF-1 plays an important role; (c) and a theory regarding immunity, stating that the immune system is programmed to decrease its functionality (immunosenescence), and this should increase the susceptibility to infectious diseases, and chronic inflammatory pathologies during aging. 

Furthermore, those theories that put the aging process under the influence of internal and external environmental factors are: (a) the theory of wear and destruction, according to which cells and tissues become worn over time by the noxious elements from cell byproducts; (b) the activity level theory—which states that a high level of basal metabolism leads to a shortening of the body’s life; (c) the cross-linking theory, assessing that the cumulative chemical exchange of important macromolecules, including collagen, causes aging; (d) the theory of somatic DNA destruction whereby aging is thought to result from the degradation of the genetic integrity of somatic cells as a result of mutations, recorded both at the nucleus and the level of the mitochondria; and (e) the free radical theory that states that superoxide and other free radicals destroy molecular components of cells and thus alter their normal functioning [3]. 

Reactive oxygen species (ROS) are probably the most important free radicals with major implications for the destruction and aging of cells and the body. Free radical theory is currently the most accepted explanation for aging, although a recent dataset was obtained on Sod2 +/− or Mclk1 +/− transgenic mice. This undermines the central dogma of the theory. In 1957, Harman advanced the hypothesis that a general process of the accumulation of oxygen free radicals negatively affects several factors in the internal environment and modifies genetic factors, the mechanism made responsible for the aging and death of all living things. The theory was revised in 1972 when it was shown that mitochondria are the main site of the chemical reactions that generate free radicals [3]. Considering aging as a gradual decrease in the functional regulation of complex multifactorial biological processes, the individual’s genotype certainly impacts on the aging rate. However, no genetic markers of the aging process have been identified, although sustained efforts have been made over the last 20 years [3]. 

The main ways to increase a healthy lifespan include lifestyle modifications and pharmacological (or genetic) manipulations [1]. Appropriate diet and caloric restriction are crucial in healthy aging [4].

Liu J.K. summarized that biogerontological studies provide a great opportunity for the pharmaceutical and healthcare industries, as antiaging medicines target enhancement of cell regeneration, autophagy induction, epigenetic change of gene activity, and restriction of calories [1].

As a matter of fact, anti-aging medicine is a relatively new medical field that is developing at a very rapid rate. This field is one of the applications of advanced scientific and medical technologies in the prevention, early detection, treatment, and cure of age-related dysfunctions. One of the main purpose of anti-aging medicine, however, is not only to prolong the life span but in particular to sustain a healthy life for a longer time. Rattan proposed shifting the approach in this field from “antiaging” to “healthy aging”, thus reinforcing health-oriented research [5]. This should explain why specialists commonly use the term healthy-aging instead of anti-aging. 

Key nutrients such as defined vitamins, minerals (as micronutrients), essential and branched amino acids, polyunsaturated fatty acids (PUFA), probiotics, and plant metabolites, such as polyphenols and terpenoids, are widely recognized for preventing aging and promoting healthy aging. Their role is mainly to counteract the oxidative stress in the body, according to the free radical theory of aging [6,7,8,9]. Older adults increase the risk of atherosclerosis caused by chronic inflammation [10]. Natural compounds can increase lifespan and improve health and quality of life by decreasing the development of some age-related chronic diseases such as diabetes, cancer, neurodegeneration, and cardiovascular illnesses [11].

The mechanisms by which oxidative stress should cause degenerative phenomena related to aging must be separated from the fundamental role exerted by ROS as signaling molecules, because they modulate and regulate important healthy and survival systems, held by mitochondria and mitochondria-associated membranes (MAM), which ensure the viability and healthy state of cells and tissues [12,13,14]. 

Antioxidants are involved in the prevention of age-related diseases such as atherosclerosis, neurodegenerative processes, cancer, diabetes, and skin wrinkles at the molecular level [3,15,16]; they also have a beneficial effect on digestion and the immune system by lowering the level of inflammatory and degenerative processes in the body. Phytoconstituents can also play a positive role in the detoxification processes of human cells [17,18,19]. Functional food, based on vegetable and fruit fibers, whole grains, nuts, seafood, and green tea, has great health potential for human health [3]. Their consumption may represent one of the healthiest and safest ways to maintain a balanced diet. 

In 2011, 30 substances with geroprotective properties were described. Among them it is worth mentioning Gerovital—this is the Romanian geroprotective product prepared by Prof. Ana Aslan in 1951 based on the anesthetic procaine, resveratrol, and other plant polyphenols, rapamycin, antioxidants, vitamins A, C, and E, carotenoids, lipoic acid, coenzyme Q, selenium, etc., hormones (GH, thyroid hormones, adrenaline and sex hormones, melatonin), bioregulatory peptides (thymaline, epithalmin), biguanide (metformin, fenformin), adaptogen (ginseng) [3]. Geroprotective/anti-aging compounds (antioxidants) studied worldwide, such as resveratrol, rapamycin or procaine, α-tocopherol, ascorbic acid, retinol, ubiquinone, selenium, etc. as endogenous compounds or as further numerous synthetic molecules in the field, interfere with the oxidative balance [3]. Natural anti-aging compounds such as vitamins, polyphenols, hydroxy-acids, polysaccharides, and many others play a crucial role in skincare [20].

This narrative review aims to highlight the role of balancing the diet through the consumption of the necessary amount of natural ingredients, including vitamins, minerals, polyunsaturated fatty acids (PUFA), amino acids, probiotics, plants’ fibers, nutritional supplements, polyphenols, some phytoextracts, and drinking water on supporting the antioxidant system of the organism and extending healthy longevity.

## 2. Vitamins

Most vitamins cannot be produced in the human body, so they are prescribed through dietary intake. 

### 2.1. Vitamin C

Vitamin C (L-ascorbic acid or L-ascorbate) is a very important water-soluble antioxidant and probably the most common hydro-soluble vitamin known so far. This vitamin is recommended for dietary intake and topical skin applications [21] as it stimulates collagen synthesis in the dermal layer and contributes to the protection against UV-induced damage [21,22]. According to the National Recommended Energy and Nutrient Intake Levels, the optimal daily intake of L-ascorbate ranges from 35 mg/d (6 months–3-years of age) to 105 mg/d (men) or 85 mg/d (women), except during lactation (130 mg/d) [23]. 

Clinical studies on the effects of differential vitamin C intake report the huge difficulty in achieving sound and reliable results [24]. It is widely known that fresh fruits and vegetables are the richest natural sources of vitamin C. 

The habit of cigarette smoking could significantly decrease the plasma concentration of vitamin C [25]. However, vitamin C deficiency does not seem to be fundamentally related to nutritional impairment [26]. Interestingly, a study on 200 patients reported that patients with hypovitaminosis C were elderly and had very high levels of inflammatory biomarkers, such as C-reactive protein (CRP), indicating that vitamin C levels decrease with age [27]. Furthermore, vitamin C could significantly affect ovarian aging in a mouse model [28].

The role of vitamin C in aging has been investigated particularly for skin health [21,29] and immunity, particularly in inflammatory and degenerative diseases [30,31,32,33].

### 2.2. Vitamin A

Vitamin A can be found in nature in two forms: vitamin A as such, also called retinol, present in a supplementary form in animal feed, and provitamin A, known as carotene, which is found in both animal and plant products [34]. Retinol is a highly effective antioxidant. Retinoids, both natural or synthetic, such as tretinoin and tarazotene, were recently introduced as possible pro-drugs for preventing skin aging, particularly for photoaging [35,36,37]. If retinoids, which represent a synthetic form of vitamin A, appear effective in preventing skin degeneration due to aging, then natural sources of vitamin A should have a leading role in this context [38]. 

The role of retinol has been so far associated only with vision. In fact, retinol plays an important role in the good functioning of the visual organs, the respective deficiency leading to the diminution of the adaptive capacity of the eyes to diffused light; in more serious cases, ulceration of the ocular mucosa and even of the cornea can occur, which can cause opacification of the crystalline sorption [39]. Moreover, retinol is recommended for β-carotene. This option is justified by the reduced toxicity of this trophin, which also prevents the onset of certain forms of cancer, lowering the level of cholesterol, and thus reducing the risk of heart disease. The role of retinol is also fundamental in hampering the senescence effects on human tissues, such as skin [38]. Even a stabilized 0.1% retinol facial moisturizer may improve skin health, as recently reported [40]. At the same time, retinol plays a role in spermatogenesis, placental, and embryonic development [41]. Finally, deficiency in Vitamin A can multiply the deficiency in Fe in the case of anemia. It has been shown that vitamin A supplementation has beneficial effects on the treatment of anemia, improving the nutritional status of iron, both in children and pregnant women. These effects are much stronger in treating anemia than if iron or vitamin A were given separately [42].

Many geometric isomers of retinol, retinal, and retinoic acid may appear due to the cis- or trans configuration of the four double bonds found in the side chain. Cis isomers are less stable and can be easily converted to the trans configuration. Some of them are found in the natural state and hold essential functions [39]. 

The 11-cis-retinal isomer is the rhodopsin chromophore, the vertebrate photoreceptor molecule. Rhodopsin is formed by covalently binding an 11-cis-retinal Schiff base to an opsin protein (with sticks, blue, red, or green cones). The vision process is based on the light-induced isomerization of the 11-cis chromophore in the all-trans, resulting in a change in the molecule’s photoreceptor, so one of the first signs of vitamin A deficiency is night blindness and low visual acuity [39]. Retinol (Vitamin A1) and dehydroretinol (Vitamin A2) are found in animal foods (eggs, milk, liver) and in mainly fortified foods such as retinyl esters. During the absorption process in the intestines, retinol is esterified with saturated fatty acids and incorporated into chylomicrons that pass into the blood lymphatically. Retinol is stored in the liver as esters. The esters, in turn, can be hydrolyzed; thus, retinol passes into the bloodstream where a specific protein transports it to the extrahepatic tissues, where specific cellular proteins are bound [39].

The β-carotene contained in fruits and vegetables is considered to be a provitamin A. Provitamin-A activity is peculiar to plant-derived carotenoids. Carotenoids are pigments that cause plants, fruits, and vegetables to be red, orange, and yellow [8]. Pumpkin, carrot, apricot, and mangos are examples of vegetables and fruits containing high doses of β-carotene. At least ten varieties of provitamins and carotene have been identified in foods. However, the most representative is β-carotene, which reaches the body through food intake and is converted into the liver in vitamin A depending on needs. The name “retinol” was thus referred to as the participation of this compound in the functions of the retina. The body can convert certain carotenoid compounds into vitamin A, such as β-carotene, α-carotene, and γ-carotene. 

The carotene content of foods is expressed in μg or mg. It can be absorbed into the intestine or transformed into the enterocytes in the retinal part, which are converted into retinol and a small amount into retinoic acid. Under the action of an enzyme called carotenoid in the liver and small intestine, carotene is converted to retinol, with 6 mg of β-carotene needed to obtain 1 mg of retinol. This low yield of transformation explains that the actions that take place in the body on the part of the ingested carotene and subjected to change and metabolization, cause two-thirds of it to be eliminated by feces (4 of 6 mg), and only one third be retained in the body. Of what is retained, one half is immediately assimilated as retinol, and the other half is stored under β-carotene as a reserve for subsequent assimilation, also in retinol form, depending on the biological needs of the organism [8]. 

### 2.3. Vitamin E

Another plant-synthesized antioxidant is vitamin E, whose main sources are nuts, grains, and extra virgin oils of olive, corn, etc. Vitamin E (α-tocopherol) is an essential nutrient derived from a plant-based lipid antioxidant and useful for all vertebrates. The function of vitamin E in preventing and reducing injury induced by ROS has been well-described and highly debated [43,44]. 

Some investigations reported that vitamin E has novel functions in radical-quenching activity, particularly in the modulation of gene expression [45]. Tocopherol can prevent UV lipid peroxidation and exhibits a very positive impact on dermal protection [46]. Tocopherols are quite widespread liposoluble substances, especially in the plant kingdom. The basic structure of tocopherols is a tocol. It is a chromanol (hydroxychroman, dihydro-benzopyran) ring that can be mono-, di-, or trimethylated and hydroxylated. The chroman is composed of a benzene ring and a pyranic heterocycle. 

The tocopherols have a phenolic hydroxyl at position six and a saturated side chain, derived from phytol (C_20_H_39_OH), bound to C2 of the heterocycle. At C2, which closes the oxide ring of the pyranic part of the nucleus, a methyl radical is attached. The compounds that have this basic structure are α-, β-, δ-, and γ tocopherols. β- and γ-tocopherols exhibit reduced vitamin activity (between 15% and 30% of α-tocopherol activity). In addition, the α- and β-trienols have reduced vitamin activity (20% and 5%, respectively). The other derivatives have no vitamin activity. The position and number of the methyl group in the benzene ring influence the vitamin action of tocopherols.

In green plants (especially grasses), the biosynthesis of tocopherol starts with phytol, which is also involved in the synthesis of chlorophyll. The absorption of vitamin E by enterocytes requires, as in the case of lipid digestion, the presence of bile salts indispensable for the formation of mycelia that allows the attack of pancreatic lipase on lipids. Orally administered tocopherol esters are subject to the action of a specific pancreatic esterase that releases tocopherol in the form of α-tocopherol, the form having vitamin activity [47]. Intestinal absorption is a passive process, which occurs at a relatively low rate, and the various isomers are embedded in chylomicrons in a non-discriminatory manner. After entering the lymphatic circulation, chylomicrons are immediately hydrolyzed by lipoprotein lipase. The tissues and muscles mainly capture the released fatty acids, showing that tocopherols can be transferred together with some fatty acids into different tissues. In addition, some tocopherols are transferred together with lipoparticles that act as surface lipid residues and can enter the HDL structure. Finally, the chylomicrons remaining together with their tocopherols are captured by the liver via a modulating receptor involving apolipoprotein E [48]. In blood, tocopherols bind 40–60% low-density lipoproteins (LDL) and 35% high-density lipoproteins. The serum concentration of tocopherols is closely related to the level of lipemia and cholesterol, around 0.6–0.8 mg tocopherols/g total plasma lipids. The share of tocopherols in the serum depends on sex, age, etc. Under normal physiological conditions, the serum vitamin E concentration in adults is between 5–16 mg/L, while in older women it can reach 9–25 mg/L. In newborns, the serum vitamin E concentration is maintained at around 5 mg/L; in premature babies, it is between 2–4 mg/L [48]. 

The role of vitamin E in aging is particularly debated [49]. As with L-ascorbate, vitamin E has also been associated with the prevention of cognitive decline during senescence, particularly in Alzheimer’s disease [50]. 

Vitamin E deficiency causes enzymatic changes such as the decreased activity of the cytochrome P450-dependent oxidase system from the microsomal fraction, increased cAMP-phosphodiesterase activity, decreased cellular respiration level, preventing the conversion of cyanobenamine into its active co-enzymatic form, etc. Hypovitaminosis E is thought to activate enzymes in the catabolic pathways: RNA-z, DNA-z, cathepsins, etc. By decreasing the degree of phosphorus incorporation into nucleic acids, hypovitaminosis E leads to the inhibition of proteinogenesis and, implicitly, cell division in several antioxidant protection systems in living organisms: pyrimidine-reduced nucleotides, thiamine acids, some proteins such as ceruloplasmin, and transferrin, specific enzyme systems (superoxide dismutase (SOD), catalase, glutathione peroxidase, glutathione reductase), vitamin C, and others [51]. 

In a series of transformations aimed at destroying free radicals, there may be synergism between vitamin E and some of these antioxidant systems. Thus, the mechanism of action of glutathione in the breakdown of lipid peroxides and derivative hydroxy acids is best studied. Glutathione peroxidase is a selenium-dependent enzyme. To observe that selenium insufficiency in food causes some symptoms characteristic of hypovitaminosis E and vice versa, may indicate that tocopherol deficiency is partially offset by slightly increased selenium intake.

## 3. Polyunsaturated Fatty Acids (PUFAs)

Essential long-chain polyunsaturated fatty acids (PUFAs) are the key nutrients to prevent aging-associated abnormalities. PUFAs are important in regulating cholesterol levels and are the precursor of prostaglandins [52,53,54]. Their role in aging has emerged in recent years and will be detailed in the following paragraphs [55,56,57].

### 3.1. Omega-3 PUFA 

Omega-3 PUFA can modulate platelet aggregation and hypertension [53] and protect against senile dementia [58]. Xie et al. revealed that the human gut microbiome partially mediates the anti-aging mechanisms of omega-3 fatty acids [59].

Fish and Calanus oils, pumpkin and sunflower seeds, and walnuts are the richest sources of omega-3 PUFA [58,60,61]. The major mechanisms of PUFA action are attenuation of inflammation via the competition with the production of arachidonic and eicosanoid acids. The importance of micronutrients, such as certain microelements, for inner and skin health, has been highlighted in experimental and clinical studies [62]. Experimental and human investigations have reported pro-cognitive and neuroprotective effects of omega-3 PUFA in aging, indicating a positive relationship between regional grey matter (GM) volume and peripheral levels of omega-3 PUFA, as well as negative relationship between cognitive deficits and dietary omega-3 PUFA levels [63,64]. These data demonstrated that increased intake of dietary omega-3 PUFA in normal aging could improve the structure and function of fronto-hippocampal GM.

Furthermore, it has been reported that low status of omega-3 PUFA in middle-aged German women (40–60 years) was associated with an elevated risk of cardiovascular diseases [65]. The omega-3 PUFA was reported as improving the contents of numerous signaling factors that contribute to plasticity, increasing campal neurogenesis even at old age, and enhancing dendritic synaptic spines. In addition, the omega-3 PUFA of elderly subjects shows anti-inflammatory effects related to improved cognitive activities that highlight its efficacy in preventing the loss of integrity and white and gray matter volume [65,66].

Recent data suggested that the plasma levels of homocysteine can affect the association between omega-3-PUFA and cognitive decline in older adults [67]. Actually, numerous reports described a close relationship between omega-3-PUFA and cognitive disorders [68,69].

### 3.2. Omega-6 PUFA 

Both omega-3 (ω-3) and omega-6 (ω-6) fatty acids are important components of cell membranes and are precursors to many other substances in the body such as those involved in blood pressure regulation and inflammatory responses. The human body is capable of producing all of the fatty acids it needs, except two: linoleic acid (LA)—an omega-6 fatty acid—and alpha-linolenic acid (ALA)—an omega-fatty acid. These must come from the diet and are called “essential fatty acids”. Both of these fatty acids are necessary for growth and healing, but they can also be used for the production of other fatty acids. For example, omega-3 fatty acids, eicosapentaenoic acid (EPA), and docosahexaenoic acid (DHA) can be synthesized from ALA, however, as conversion is limited, it is recommended to also include these sources in the diet. The fatty acids ALA and LA are found in vegetable oils and seed oils. Although LA levels are generally much higher than those of ALA, canola and walnut oil are excellent sources of the latter. The fatty acids EPA and DHA are found in fatty fish (e.g., salmon, mackerel, herring). Arachidonic fatty acid (AA) can be obtained from animal sources, such as meat and egg yolks.

Recent data on *C. elegans* reported that the anti-aging effects of omega-6-PUFA may be associated with autophagy and hence with a resistant phenotype to starvation [70,71]. Plasma circulation of linoleic acid accounts for the beneficial role of omega-6-PUFA [72]. This introduces the fundamental concept that a correct ω3/ω6 ratio must be observed in order to reduce possible noxious effects due to omega-6-PUFA excess in intake [73,74]. 

## 4. Trace Elements and Micronutrients

### 4.1. Zinc 

Zinc, copper, and selenium play a vital role in maintaining body health [75]. Zinc is an important cofactor of many metalloenzymes and is able to bind to more than 300 enzymes and more than 2000 transcriptional factors [76]. Many aspects of cellular metabolism are zinc-dependent. Zinc plays an important role in growth, development, immune response, neurological functions, and reproduction. One of its main functions is to protect the skin against excessive UV irradiation [77,78]. At the cellular level, zinc functions can be divided into three categories: (i) catalytic functions; (ii) structural functions; and (iii) regulatory functions. 

Catalytic role: The ability to catalyze vital chemical reactions of approximately 100 different enzymes depends on zinc. Structural role: Zinc plays an important role in protein structure and cell membranes. The structural protein motif called a “zinc finger” (zinc finger), characteristic of a large number of receptors and transcription factors, is well known. For example, copper is located in the catalytic center of the enzyme with the antioxidant role of copper–zinc superoxide dismutase (CuZn-SOD). At the same time, it plays a structurally critical role [76]. 

The structure and functions of cell membranes are also affected by zinc. It has been observed that a decrease in zinc concentration increases the sensitivity of membranes to oxidative damage, which involve their functions. Regulatory role: Zinc finger proteins have been found to regulate gene expression by acting as transcription factors (they recognize specific sequences in the DNA structure and influence the level of transcription of specific genes). Zinc also plays an important role in cellular signaling, influencing the release of hormones and nerve impulse transmission. Recently, it was discovered that zinc plays a role in apoptosis (programmed cell death), a process of critical cellular regulation with implications for growth and the development of several chronic diseases [79]. The bioavailability of zinc (the amount of zinc retained and used in the body) is relatively high for meat, eggs, and marine products. This is due to the relative absence of the compounds that stop the absorption of zinc and the presence of certain amino acids (cysteine and methionine) that improve zinc absorption. Zinc from whole grains and plant protein products is less bioavailable due to its high amount of phytic acid, a compound that inhibits zinc absorption. The enzymatic action of yeast used in bread-making reduces the level of phytic acid. As a result, whole-grain bread contains more bioavailable zinc than non-whole-grain bread. Recent statistical surveys on eating habits in the U.S. estimated that the average daily dose of dietary zinc is 9 mg/day for adult women and 13 mg/day for adult men [80]. 

Zn is also required for activation of the enzyme that catalyzes the transformation of retinol into the retina. At present, the effects of Zn deficiency on the nutritional status of vitamin A are not exactly known. However, Zn deficiency is known to interfere with vitamin A metabolism in several ways, causing: (i) decreased retinol transporter protein (RBP) synthesis; (ii) decreased activity of the enzyme that releases retinol from its hepatic storage form (retinyl palmitate).

### 4.2. Copper

Copper is known to stimulate the maturation of skin collagen as a key component for improving skin elasticity [81]. Copper from food is absorbed into the stomach and proximal parts of its small intestine. This process takes place under anaerobic conditions and is energy-dependent. The degree of absorption is 10% in animals and 32% in humans and depends on the chemical form found in food and the pH of the intestinal contents. Studies with the ^64^Cu isotope revealed that after oral administration, the concentration of copper in the blood reaches a maximum threshold after 0.5 h, and the absorption rate is influenced by copper’s ability to bind to L-Ala, with which chelate complexes are formed. Organic compounds can affect the absorption of copper from food. 

Thus, in large quantities, phytates and ascorbic acid decrease the absorption rate. The copper route does not flow in a closed system, so the main direction of copper transport is to the liver to synthesize ceruloplasmin. It is secreted into the plasma, exerts its oxidase activity, then is captured by the liver, degraded, and eliminated by the bile. Although it represents 4% of the concentration, the labile form fixed to the albumin ensures the transport of plasma copper to tissues, having a rapid turnover. It has been established that over 96% of the ingested copper is disposed of in this way. Urinary elimination is a very small fraction (below 1% of ingested copper), a phenomenon that can be explained by fixing copper to proteins.

### 4.3. Selenium 

Selenium is a component of selenoproteins that contributes to the alleviation or reduction of inflammation, DNA damage, and prolonged telomere length and thereby plays a role in fighting age and preventing cardiovascular diseases, neuropsychiatric disorders, tumors, and skin aging, among others [82,83]. So far, at least 11 selenoproteins have been characterized, and there is evidence that their number is higher. 

Glutathione peroxidase (GPx): Four such enzymes are known to be selenium-dependent: classical cellular glutathione peroxidase (GPx); extracellular GPx, or plasma; phospholipid hydroperoxide GPx; and gastrointestinal GPx. Although each GPx is a distinct selenium-dependent enzyme, all have an antioxidant function by reducing ROS, such as peroxide ions and lipid hydroperoxides, by coupling the reduction reaction with the glutathione oxidation reaction. Selenoprotein from sperm capsule mitochondria, an antioxidant enzyme that protects sperm from developing oxidative damage and which later forms a structural protein required for mature sperm, was considered a distinct selenoprotein, but subsequently proved to be a phospholipid hydroperoxide GPx. Thioredoxin-reductase: together with thioredoxin, this enzyme participates in the regeneration of several antioxidant systems, including vitamin C. 

Maintenance of thioredoxin in reduced form by thioredoxin-reductase is important for regulating cell growth and viability [84]. Iodothyronine-deiodinase (thyroid hormone deiodinase): The thyroid gland releases in the blood very small amounts of active thyroid hormone (triiodothyronine = T3) and larger amounts of its inactive form (tetraiodothyronine = thyroxine = T4). Most T3 is synthesized by removing an iodine atom from T4 through a reaction catalyzed by selenium-dependent iodothyronine-deiodinases. There are three known iodothyronine dyskinesias designated I, II, and III, which, by their action on T3, T4, and other metabolites of the thyroid hormone, can activate/inactivate the thyroid hormone, making selenium an essential element in the normal development and growth as well as in the metabolic pathways controlled by thyroid hormone [85]. Seleno-protein P is found in plasma and is associated with endothelial vascular cells at the level of the internal wall of blood vessels. 

Although the functions of selenoprotein P have not been fully elucidated, it is assumed to be a transporter protein, an antioxidant capable of protecting the endothelial cells from attack by reactive nitrogen species (NRS) called peroxynitrites. Seleno-protein W: this is found in muscles. Although its function is unknown, it is thought to play an important role in muscle metabolism. Selenophosphate synthetase: this is the enzyme that catalyzes the reaction of incorporation of selenocysteine into selenoproteins. This protein catalyzes the synthesis of selenium monophosphate, a selenocysteine precursor that is required to synthesize selenoproteins. 

Being an integral part of GPx and thioredoxin reductase, selenium interacts with each nutrient, affecting each cell’s pro-oxidant/antioxidant balance. It is assumed that selenium from GPx is an activator of vitamin E in lipid peroxidation. Studies in laboratory animals have shown that selenium and vitamin E protect each other. It appears that selenium may prevent some of the disorders that occur as a result of vitamin E deficiencies. In addition, thioredoxin reductase maintains the antioxidant functions of vitamin C, catalyzing its regeneration reactions. 

Selenium deficiency can counteract the effect of iodine deficiency. Iodine is essential for thyroid hormone synthesis, but selenozyme deiodinases are also required for T4 to T3 conversion. Selenium supplementation of the elderly diet decreases the concentration of T4, indicating that an increase in deiodinase activity may increase the conversion rate of T4 to T3 [86,87].

### 4.4. Role of Zn, Cu and Se in the Aging Prevention 

The role of Zn in aging and immunosenescence has recently been reviewed [78]. In fact, Zn, which is often deficient in the elderly, rules many functions characterizing the so called “oxi-inflamm-aging”, at least because of the aforementioned functions at the biochemical level [78,88]. A proper plasma level of trace elements, such as Zn or Cu, promotes an optimal function of the immune response [75]. High levels of copper, for example, have been associated with cognitive impairment [89]. In this context, the role of micronutrients is particularly crucial [90].

For example, Se is a fundamental cofactor in many redox functions, which reduces the ROS-induced degeneration in the senescent phenotype [91]. Cofactors of major enzymes involved in the clearance of oxidative stressors are surely beneficial in preventing aging-related damages [90]. In the case of Zn, its role in the optimal function of the immune responses appears particularly crucial, as old mice with reduced zinc levels reported an increased proinflammatory cytokines profile, such as MCP1 and IL6 in the serum, also showed increased Th1/Th17/inflammatory cytokines (IFNγ, IL17, TNFα, respectively), and decreased naïve CD4 T-cells in the mesenteric lymph nodes (MLN) [92]. The ability to enforce immunity in the elderly and to delay the immunosenescence mechanism is a leading role of these trace elements, usually in the form of metal cofactors [93,94,95,96].

## 5. Carnitine

Amino acids are the components of proteins that significantly contribute to aging-related diseases [97]. Perspective natural UV-absorbing compounds for skin protection are mycosporine-like amino acids, which can absorb UV radiation and disperse the absorbed energy without generating reactive oxygen species [98].

However, dietary intake of an excessive amount of a single amino acid can be toxic [97]. The multifunctional anti-inflammatory carnitine, and its acetyl derivative l-carnitine are amino acids that play an essential role in fatty acid transport within the mitochondria that convert metabolic pathways into energy. Carnitine is a non-essential amino acid that plays a role in the transport system. In fact, it binds the free fatty acids in the cytoplasm to cross the two membranes of the mitochondria to be later oxidized; thus, it is not an enzyme but a transport system.

The main function of carnitine is the transfer of long-chain fatty acids (LCFA) to mitochondria for subsequent β-oxidation. This process provides biological energy, starting from a lipid substrate, not glucose. The advantage is that simultaneous fat loss occurs through the preferential use of lipids. Carnitine is found on the pharmaceutical market and as a nutrition product (vials with oral carnitine) for athletes. As a non-essential amino acid, carnitine is not prohibited by sports ethics; moreover, a diet rich in animal proteins brings significant quantities of carnitine into the body. For this reason, carnitine is a good adjuvant of local anti-cellulite treatment and physical culture. It stimulates the formation of muscle mass by reducing fats.

## 6. Plant Metabolites

Various plant metabolites derived from polyphenols, triterpenes, and sterols classes demonstrated promising antioxidant and anti-aging effects [99,100]. Plant-derived antioxidants, both by topical and oral applications, may prevent free radicals’ over-production and, therefore, different diseases caused by oxidative stress and redox-derived stressors, including aging [17,101]. Secondary phytoconstituents include polyphenols (stilbenes, anthocyanins, epigallocatechin gallate, curcumin, rosmarinic acid, flavonoids, etc.), and play a significant role in limiting aging processes in the body and skin due to the ability of OH-groups to inhibit the influence of free radicals [18,102,103,104]. After the transdermal application of rosmarinic acid, its inhibitory effects on collagenase, elastase, and antioxidant activities were proved [105]. Blueberry anthocyanins effectively protect against the aging process in the epithelial cells [106]. Quercetin improved the spatial learning and memory impairment of aging mice [107]. A high level of polyphenols such as epigallocatechin gallate, catechin, rosmarinic acid, flavones, etc., evaluated in green tea and some medicinal herbs, is the key to their antioxidant potential [108,109,110]. Dietary consumption of flavonoid-rich foods and nutraceuticals can improve cognitive function and inhibit senescence [111]. Increasing the consumption of phytosterols may be an important way to reduce cholesterol levels and prevent coronary heart disease, cancer, wrinkles of the skin, etc. [112].

### 6.1. Polyphenols

Multiple polyphenols have substantial health-promoting effects as they are powerful antioxidants [113]. Flavonoids are components in many fruits and vegetables containing yellow, red, or blue pigments; they have an antioxidant activity, similar to vitamin C, and an anticarcinogenic action [114]. 

Polyphenols are some of the most potent antioxidants in food. There are over 8000 polyphenolic structures identified in plants and these compounds being present mainly in fruits, vegetables, tea, wine, cocoa, aromatic plants, or coniferous bark [115]. Polyphenols can be in the form of anthocyanins (in red fruits), flavonoids (in citrus fruits), quercetin (in tea leaves, cocoa, onions, algae, apples), resveratrol, stilbenes (in grapes, pomegranates), lectins (in legumes), and lignans (in flax seeds) [106]. 

Chemically, they contain one or more aromatic nuclei, on which several hydroxyl groups are grafted, and four main classes of polyphenols are known: flavonoids (cvercetol, kaempferol, luteolin, genistein, simple and condensed catechins, proanthocyanidins, the latter being commonly classified in the category of tannins), phenolic acids (chlorogenic acid, rosmarinic acid, ferulic acid), stilbenes (trans-resveratrol), and lignin derivatives (pinorezinol). Plant polyphenols are one of the most important categories of natural active principles with antioxidant effects, being increasingly used in skin antiaging therapy, having remarkable properties in combating the harmful effects of solar radiation and atmospheric pollutants, preventing and alleviating the symptoms of premature skin aging. Specifically, polyphenols are recognized for their antioxidant, anti-inflammatory, antibacterial, antiviral, antitumor, and anti-atherogenic action (against deposition on blood vessels) [116,117]. The main action of antioxidants is to prevent the formation of free radicals, fighting against aging processes. Polyphenols are especially useful for the plants in which they are found. Plants use them to protect themselves from parasites and microbes, from ultraviolet rays, and to favor the pollination process. Then, once they reach the human body, the polyphenols intervene to protect the cells from oxidative stress, inflammation, and genetic mutations.

Experimental studies have shown that at topical application, polyphenols can combat some clinical and histological changes in the epidermis and dermis induced by exposure to UV radiation and chronological aging, causing the restoration of the keratinocyte ultrastructure, stimulation of collagen synthesis, improvement of vascularization or normalization of hyper-keratinization, being able to function in different phases of aging, as this is a stepwise process. Each phase or clinical form involves a specific treatment. Plant species with a high concentration of polyphenols of cosmetic interest include *Vitis vinifera* (vines), *Punica granatum* (Rodia), and *Camellia sinensis* (tea). The fruits of *Vitis vinifera*, the grapes, have anti-aging activity at the skin level due to their content in proanthocyanidols and trans-resveratrol, showing protective effects against oxidative stress induced by UV radiation mainly by supporting the functionality of endogenous antioxidant systems, preventing (photo) macromolecular biological degradation (lipids, proteins, DNA) and inhibition of the activation of cellular signaling pathways MAPK (mitogenically activated protein kinase) and NF-kB (nuclear factor kappa B), involved in photocarcinogenesis. 

Pomegranate polyphenols (flavonoids, proanthocyanidols, and, in particular, punicalagin-type ellagitannins) exhibit antioxidant, anti-inflammatory, and antiproliferative action, and DNA regeneration capacity, resulting in photoprotection and photo-chemoprevention. Phenolic acids are simple molecules easily absorbed into the human system and offer several anti-aging benefits. In addition to making cells stronger and resistant to degradation, the anti-aging property of phenolic acids is correlated with antioxidant activity that also prevents abnormal cell growth. 

Phenolic acids are also useful in controlling inflammation, stimulating the immune system, limiting the collagen fiber breakdown by various mechanisms, helping to form natural collagen fiber bonds and preventing them from being caused by free radicals, and improving circulation to the blood; all of these together produce significant anti-aging benefits in the body [113]. The antiaging skin effect is achieved by inhibiting morphological changes in the fibroblasts, stimulating the expression of type I procollagen, inhibiting MAPK, and NF-kB activation, inhibiting UVB-mediated cell proliferation, and reducing epidermal hyperplasia induced by UVB radiation and leukocyte infiltration. 

A major source of inflammatory reactions and oxidative stress, inhibition of matrix metalloproteinase activity (MMP-1, -2, -3. -7, -9, -11, -12), important in the degradation of extracellular and photo-aging matrix components, and also by inhibition of COX-2 cyclooxygenase and inducible nitric oxide synthase, enzymes involved in the processes of skin inflammation and cell proliferation. 

The flavonoid, 4,4′-dimethoxychalcone (DMC), which occurs naturally in the Ashitaba plant, induces a process called autophagy (a process of cleaning and recycling). DMC has been investigated as an anti-aging compound with cardioprotective effects in mice and can potentially promote longevity among species. For example, when Dasatinib (a leukemia drug) and quercetin (a natural product found in vegetables) are combined, improved health and prolonged life are observed [118,119,120,121].

Tannins represent the group of water-soluble phenolic compounds. The tannins in grapes are responsible for the astringent taste and body of the wines. Tannins in grape seeds help lower LDL (low-density lipoproteins) and VLDL (very low-density lipoproteins) cholesterol and increase HDL (high-density lipoproteins). Tannins reduce the intestinal absorption of cholesterol and improve bile excretion; thus, bile salts bind to cholesterol and tannins, which are eliminated through feces. This mechanism of eliminating cholesterol from the intestinal lumen seems similar to dietary fiber intake. 

The astringent action is highlighted on mucous membranes and tissues. The mechanism of action is explained by the coagulation of proteins that have a retracting effect by shrinking the lesion’s surface. A precipitation reaction of the microorganism explains the antiseptic action; it protects the wound from infections. The hemostatic action is defined by the precipitation of red blood cells. The astringent action explains the antidiarrheal step. They are used as an antidote, especially in alkaloid poisoning. In therapeutics, they are used for anti-irritating, anti-inflammatory, bactericidal, hemostatic, mild local anesthetic, and reducing secretions’ effects [122].

#### 6.1.1. Resveratrol 

Resveratrol is a polyphenol of the stilbenoids group detected in high quantities in grapes’ skin, seeds, and red wines. This phytoalexin possesses a very promised antioxidant potential [123,124,125]. Resveratrol may extend the lifespan of humans by activating the SIRT1 and sirtuins’ molecules. Sirtuins are a class of enzymes that control cellular metabolism by regulating the expression of certain genes. It is known that resveratrol is a sirtuin 1 activator, represented by the SIRT1 gene, improving mitochondrial function and slowing the proliferation of certain cancers. This gene also controls the longevity of several species of animals, including the longevity of humans.

Resveratrol could inhibit apoptosis and morphological modifications stimulated by H_2_O_2_ treatment, increase proliferation, and reduce acetylated TP53 [126]. Resveratrol (found in red grape bark, *Polygonum cuspidatum*, groundnuts, blueberries, and other berries) is a chemical compound that some plants synthesize to remove bacteria and fungi, as well as to protect against ultraviolet (UV) radiation. 

Preclinical studies on resveratrol have shown an increase in longevity in *S. cerevisiae* by 70% by cultivation on a medium containing 10 mM resveratrol, 20% of *C. elegans*, 29% of *Drosophila melanogaster* by treatment with 100 mM resveratrol. In studies in laboratory mice, resveratrol in doses around 20 mg/kg resulted in a statistically significant decrease in age-related parameters: albuminuria; inflammatory levels; vascular endothelial apoptosis; decreased aortic elasticity; cataract incidence, etc., being recorded, including data on reducing genetic instability [106]. 

Resveratrol protects against Alzheimer’s disease by blocking the NF-Kb protein, thus preventing microglia from destroying neurons. There are encouraging studies regarding the therapeutic potential of resveratrol and other neurodegenerative diseases. 

Resveratrol has also been beneficial for exercise and physical and mental performance. Resveratrol’s effects, similar to phytoestrogen hormones, are very promising: it is the most powerful calcium fixator in bones (in addition to physical exercise), effectively preventing and combating osteoporosis; it confers major vascular protection, even in men; combats climacteric disorders; helps to regulate the menstrual cycle and cancels the effects of dysmenorrhea; even in excess, it provides protection against breast and cervical cancer and does not induce cancer; protects the nerve cells against the devastating effects of stress, prolongs their life and prevents their apoptosis; promotes the renal elimination of uric acid, preventing its accumulation and the risk of degeneration in gout or uric lithiasis; increases the synthesis of endogenous antioxidants, of the substances needed by the body (phytoestrogens are enzyme-inducing substances—hepatic—with a role in detoxifying the body). 

In MRC5 human fibroblast cultures, 5 μm resveratrol induced significant protection against oxidative DNA damage, preventing increased nuclear volume, reduced generation of acetylated forms of histones H3 and H4, and p53 protein. In another study on human fibroblasts, the use of a concentration of 10 µm and 25 µm led to data that supported the delay in the appearance of morphological changes at the cellular level correlated with age [106]. Resveratrol-like polyphenols are supposed to inhibit cellular senescence by activating p53 and AKT genes, sirtuins or inhibiting others, such as mTOR. They influence different intracellular signaling pathways by which the expression of genes involved in cell growth, proliferation, and cell viability are controlled. Clinical studies on the action of resveratrol in oncology using a commercial form of resveratrol called SRT501 showed a 39% increase in malignant cell apoptosis in patients with metastatic colorectal cancer [106]. 

The neuroprotective effects of resveratrol have been described experimentally in laboratory mouse studies. They have also been explained by researchers through the effect of growth resveratrol on the level of cysteine that can protect cells from oxidative stress by controlling the protein precursors of the amylase plate. Resveratrol also acts on manganese superoxide dismutase (MnSOD), a group of enzymes that degrade the generated superoxide molecules metabolic, therefore having an antioxidant effect [106]. The cardioprotective effect of resveratrol and other polyphenolics such as quercetin or catechins have been observed in *in vitro* studies showing a reduction in cardiomyocyte apoptosis by lowering caspase-3 levels and other cytokines, including NF-kB2, E selectin, troponin, or TNF-α [127].

Resveratrol also has an anti-inflammatory effect, leading to decreased cyclo-oxygenase activity, with a key role in synthesizing other cytokines such as IL17. The hypothesis of the antidiabetic use of resveratrol is explained by the activation of SIRT1 and subsequent increase in insulin sensitivity, improvement of microcirculation, and peripheral nerve function [128]. Resveratrol also acts on the cellular mechanisms involved in photo-aging correlated with the action of UV rays, including MAP kinases, nuclear factor NF-kB and matrix metalloproteins. External applications of resveratrol to the SKH-1 hairless mouse model before ultraviolet exposure resulted in a significant reduction in cell proliferation, mRNA protection, and phosphorylation. However, the pharmacology of resveratrol is marked by several limitations: low water solubility and consequently low bioavailability, stability, and is easily oxidizable in the presence of light or heat. Even the data contradict that resveratrol would result in the extension of longevity, obtained in research on *Drosophila melanogaster* and *C. elegans* and disseminated by some authors [129].

#### 6.1.2. Curcumin 

Curcumin can positively slow down aging by suppressing age-related changes in the inflammatory processes [130]. Research on aging and related traits of curcumin in model organisms has reported that curcumin and its metabolite, tetrahydro-curcumin (THC), could increase the mean lifespan of at least three investigated model organisms such as the fruit fly *Drosophila*, mouse, and nematode roundworm. 

A significant elevation of lifespan could be seen by decreasing the production of reactive oxygen species by genes (skn-1, sek-1, osr-1, mek-1, sir-2.1, unc-43, and age-1) in the nematodes’ models grown on enriched media with curcumin. The extension of lifespan of *Drosophila* by curcumin supplementation was related to declined malondialdehyde and lipofuscin levels and increased superoxide dismutase activity (SOD) [131]. Curcumin also reverses endothelial dysfunction and artery stiffness and may be a novel therapy for treating arterial aging in humans [132]. Curcumin is currently used to treat numerous disorders, particularly those that contribute to an inflammatory process. Curcumin and its derivatives have been reported to have a powerful anti-cancer function, particularly on cancer stem cells (CSC) [126].

### 6.2. Terpenoids 

Due to terpenoids, essential oils showed their valuable anti-aging potential in treating anxiety, dementia, and other neurological disorders via *in vitro*, *in vivo*, and clinical studies [111,133]. Essential oils are useful as multi-potent agents due to their composition of several valuable components [134,135]. Aromatherapy with essential oils can improve cognitive performance in patients with Alzheimer’s disease [133,136]. The use of essential oils and essential oil-bearing plants can provide significant benefits to health through cognitive improvements. 

A combination of aromatherapy by topical, inhalation, or ingestion mode of application can enhance the positive effect on the human body [133,136]. Molecules of terpenoids are small and can be transferred across nasal mucosa during inhalation, enter into the blood and penetrate through the blood–brain barrier, or cross through the skin after topical application due to fat-soluble properties [133]. Ursolic acid, a pentacyclic triterpenoid in many fruits and herbs used in daily life (apples, prunes, cranberries, elder flower, peppermint, rosemary, holy basil, bilberries, etc.), has hepatoprotective properties to enhance the anti-aging biomarkers [137].

### 6.3. Other Natural Compounds from Medicinal Plants

Usually, many plant-derived phenolic and polyphenolic substances, which include the commonest flavonoids (isoflavones, lignans, flavones and so forth), behave as phytoestrogens, namely compounds with a hormonal-like function, usually on estrogen receptors. Not all of these compounds act yet by targeting estrogen receptors. Studies on these compounds have demonstrated their efficacy in preventing carcinogenesis with localization to the mammary, prostate, and colon glands and preventing osteoporosis. 

Many kinds of vegetables, when eaten raw, have substantial anti-aging effects because they slow down the development of Alzheimer’s disease [138]. Most of them are the representatives of botanical families such as *Amaryllideae* (onion, garlic), *Apiaceae* (carrot, parsley, dill), *Asparagaceae* (asparagus), *Brassicaceae*(red cabbage, broccoli, radish), *Cucurbitaceae* (cucumber, pumpkin), *Fabaceae* (soybean, red bean), *Dioscoreaceae* (Yam), *Amaranthaceae*(Chinese Spinach), *Asteraceae* (Artichoke), etc. 

Medicinal plant sources include, amongst others, seaweeds, *Allium sativum*, *Cynara scolymus*, *Crataegus* spp., *Ginkgo biloba*, *Hippophae rhamnoides*, *Panax ginseng*, *Schizandra chinensis*, *Silibum marianum*, and *Aloe vera*, representatives of which are very useful in the prevention of aging-related ailments [6,17,139]. For instance, the common application of the *Allium sativum* extract and coenzyme Q10 showed a favorable effect on inflammatory parameters and atherosclerosis progression [140]. S-Allylcysteine, an organosulfur molecule from aged garlic, can ameliorate the aging process via regulating mitochondrial functions [141].

Diallyl sulfide, also found in garlic, has also been found to aid the elimination of arsenic from the body proving effective in ameliorating arsenic toxicity [142,143].

*Silybum marianum* seed oil effectively decreased oxidative damage, and improved mitochondria function in the liver of aging mice [144]. 

*Ginkgo biloba* leaf extracts are widely used in the treatment of various degenerative diseases such as cerebrovascular disorders, Alzheimer’s disease, skin aging, etc., due to their ability to prevent mitochondrial dysfunctions and apoptosis [145].

### 6.4. Alpha-Hydroxy Acids (AHAs)

These are plant- and animal-derived substances used in various skincare products. Alpha-hydroxy acids (AHAs) such as citric acid (CA), glycolic acid (GA), lactic acid (LA), malic acid (MA), and tartaric acid (TA), are the naturally-occurring organic acids found in many fruits, berries, and herbs [146,147,148,149]. They are mostly used to reduce the impact of acne and enhance dry and aging skin. They help remove the top dead epidermal layers and promote the firmness of the deeper layers of the skin [149].

There are seven types of AHAs commonly used in products usable everywhere in the mass-production of skincare. These are the following compounds and sources: the already mentioned citric acid (from citrus fruits), glycolic acid (from sugar cane), hydroxycaproic acid (from royal jelly), hydroxycaprylic acid (from animals), lactic acid (from lactose or other carbohydrates), malic acid (from fruits), and tartaric acid (from grapes). Out of all of the AHAs available, glycolic and lactic acids have the most trusted sources and which have been most studied. However, both glycolic and lactic acids can irritate [146]. 

AHAs are first and foremost used to exfoliate. AHAs have important implications for lightening the complexion, correcting discoloration from scars and age spots, improving the appearance of surface lines and wrinkles, increasing product absorption, preventing acne breakouts and promoting collagen and blood flow [146]. Another acid particularly used in the skincare market is beta-hydroxy acid (BHA). In contradistinction to AHAs, BHAs are mostly synthesized from one source: salicylic acid (an acne-fighting ingredient) [146]. The studies evidence that their advantages extend far beyond exfoliation. AHAs facilitate the synthesis of glycosaminoglycan and collagen production and enhance the number of elastic fibers.

### 6.5. Phytoestrogens

Phytoestrogens represent a heterogeneous group of non-steroidal compounds found in plants naturally, and due to their molecular structure being similar to estradiol (17-estradiol), they can mimic its effects in the body. Estrogenic compounds widespread in herbs (garlic, parsley), wheat (soy, rice), vegetables, fruits, and coffee. They play an essential role in plants because they are part of their fungal defense system. Once ingested by humans, they bind to estrogen receptors and produce many effects; but they cannot be considered nutritients. They do not participate in any essential biological process, and their lack in the diet does not lead to any specific deficiency syndrome. 

The most important classes of phytoestrogens are isoflavones (genistein, daidzein, glicetin, formononetin, biochanin A, and equol, an isoflavone metabolite), coumestans (coumestrol), flavonol (quercetin, kaempferol), and lignans (enterolactone, enterodiol). The first three classes are better known in the medical world as flavonoids [117]. They have a more intense estrogenic effect than the class of lignans, and the antioxidant effect is particularly strong for all types of compounds. The lignans are, for example, enterodiol and enterolactone found in whole grains, fiber, seeds, and fruit and vegetables, and isoflavones such as genistein and daidzein appear in soy and other vegetables. Linseed phytoestrogens not only inhibit estradiol production, as do drugs used in hormone-inhibiting chemotherapies, but also boost estradiol metabolism in a positive direction by generating a larger amount of 2-hydroxyestrone metabolite instead of less beneficial 16- hydroxyestrone [150]. Phytoestrogens have known neuroprotective properties, including prevention of the formation of amyloid plaques and preservation of ATP depletion, most likely by inhibiting the neurotoxic effect of glutamate, as shown on rat PC12 cell cultures. The anti-atherogenic effects of phytoestrogens have been confirmed by recent studies on LDL oxidation and inhibition of superoxide radical generation, thus having antioxidant properties that interfere with the cellular and molecular mechanisms of aging [151].

### 6.6. Cocoa Derivatives 

Chocolate, a major cocoa derivative, is considered the key to an everlasting memory. Chocolate with an increased cocoa content (minimum 70%) is an excellent source of flavonoids, compounds that help improve blood flow to the brain. In fact, epicatechin, a flavonoid presents in dark chocolate, and also in berries, tea, cocoa, possesses a significant antiaging effect [152].

In general, consuming smaller amounts of chocolate at more frequent intervals seems preferable to ensure a more stable flow of nutrients into the bloodstream. There is a big difference between milk chocolate, which generally contains a lot of sugar or sweeteners, and chocolate called “therapeutic chocolate” [153]. To better understand this, here are some details about chocolate ingredients. 

Cocoa refers to the Theobroma cocoa plant, grown for its seeds, known as cocoa beans. Cocoa is naturally rich in antioxidants and other natural compounds that benefit cardiovascular health and control body weight. In total, about 40 different health benefits were highlighted in the case of regular consumption of dark chocolate. Cocoa powder refers to the powder obtained from roasted and ground cocoa seeds; generally, this variety does not contain fats. Cocoa butter is the fat component of cocoa beans. 

Cocoa butter contains unsaturated fats, omega-3, omega-6, and vitamins A, E, and K. In addition, cocoa butter is the basic component of any quality chocolate. The antioxidants it contains fight against the free radicals responsible for skin aging. Chocolate is the solid or sweet food from cooking cocoa beans (usually fried). If the cocoa beans are not fried, then raw, unprocessed chocolate is obtained, which is usually sweetened. Generally, the more concentrated the chocolate, the more that its antioxidant content increases [154]. 

Milk chocolate has little or no health benefit, as it contains limited quantities of cocoa. Cocoa powder is normally quite bitter and differs from chocolate sweetened with refined sugar, which is the most consumed. Nutritionists consider certain dark chocolate or raw cocoa powder types to be superfoods, among foods rich in antioxidants and anti-inflammatory substances. Many recent medical researchers have focused on how cocoa powder (and dark chocolate) benefits the heart and blood vessels.

These benefits appear to largely depend on the action of beneficial bacteria in the gut [155]. Cocoa powder is rich in powerful antioxidants polyphenols. It was previously considered that these molecules were difficult to digest and absorb due to their size. However, certain bacteria in the gut break down and ferment the components present in dark chocolate, turning them into anti-inflammatory compounds readily absorbed by the body. In particular, beneficial microbes, including Bifidobacterium and lactic acid bacteria, like to feed on cocoa. These beneficial microbes also break down the fibers found in cocoa powder, transforming them into short chains of fatty acids that are well absorbed by the body and give the sensation of satiety [156]. This study could explain why dark chocolate is so good for the heart, because anti-inflammatory compounds can reduce the inflammation of cardiovascular tissue. Possible explanations are: “The fibers in dark chocolate, for example, are fermented, and the large polyphenolic polymers are metabolized into smaller molecules, which are more easily absorbed. These smaller polymers exhibit anti-inflammatory activity. When the body absorbs these compounds, it helps decrease the inflammation of the cardiovascular tissue, which reduces the long-term risk of stroke”.

Other research has also shown that regular consumption of dark chocolate can help good bowel health, selectively feeding beneficial bacteria instead of harmful ones. It seems that dark chocolate acts as a probiotic, thus helping to maintain healthy gut flora. In general, the darker the chocolate, the higher the cocoa content. However, natural cocoa is quite bitter, and the higher the percentage of cocoa, the more bitter the end product will be. Flavonoids are the bittersweet taste of chocolate, but they are responsible for the many health benefits of dark chocolate. However, it should be considered that restricting calories is also crucial in the metabolic control of longevity [157].

### 6.7. Humic Substances 

*Humic substances* (HSs) are natural organic substances yielding 50 to 90% of the organic matter of peat, lignites, sapropels, and the non-living organic matter of soil and water ecosystems. HSs are naturally occurring heterogeneous organic compounds characterized as being yellow to black with a high molecular weight. Based on solubility, HSs are divided into three fractions: humic acids (HAs) that are insoluble in water under acidic conditions (pH ˂ 2) but soluble at higher pH values, fulvic acids (FA) that are soluble in water under all pH conditions and humin, that is the fraction of HSs that is insoluble in water at any pH value [158,159]. 

HSs are redox-active macromolecules that play significant roles in pollutant redox reactions and have gained great interest [160]. HSs are currently considered a promising carrier agent in drug-delivery systems due to their ability to increase the biological activity of main ingredients and nano- or microparticles [161,162].

The composition of HAs varies according to the origin, methods of obtaining them, and occurrence of different biologically active principles (quinones, phenols, and carboxylic acids). Quinones are responsible for ROS production in HAs and possess wound healing, fungicidal, and bactericidal effects. The development of antioxidant and anti-inflammatory action of HAs is due to the content of phenols and carboxylic acids. The occurrence of phenolic groups in HAs ensures antioxidant effects due to their free radical-scavenging activity [161]. Antioxidant properties exhibited by humic substances and their fractions have been demonstrated [160,163,164,165,166].

Antioxidant effects of HSs, shown by *in vitro* enzymatic luminescent bioassay, allowed the recommendation of them as a natural detoxicant [167]. Khil’ko et al. evaluated the antioxidant capacity of HAs from brown coal. They verified that the oxygen absorption rate decreased significantly in the presence of Has, and at high concentrations (10 g L^−1^), the oxidation process was completely halted [168]. 

The antiaging effects of fulvic acid for elderly patients have been demonstrated in clinical studies in China and India. The administration of fulvic acid resulted in managing dementia symptoms, better appetite, sleeping, and higher performance [162,169,170].

FA exhibits chelating effects and impacts the *in vivo* treatment of eczema. It is noted that the fibroblasts and matrix metalloproteinases are responsible for collagen degradation. A study by Kinoshita et al. demonstrates the likelihood of an anti-aging effect of FA is due to an increase in the vitality of fibroblasts and avoidance of collagen degradation [171]. FA administrated externally improved skin conditions [172].

Humic extracts were able to prevent tumors of the esophagus. In the case of thyroid tumors, HSs injections were found to be a highly effective agent. HAs injections inhibit growth and reduce the sizes of thyroid carcinoma cells [170]. The cytotoxic effect of HAs on human breast adenocarcinoma MCF-7cells has been established [173]. 

Complexes of β-carotene and HAs were synthesized by Martini et al., providing an increase in β-carotene solubility in water and stability towards light irradiation [174]. Carotenoids are precursors of vitamin A, possessing potent antioxidant capacity. FA and HA were proposed as delivery systems for other poorly soluble active principles [175,176]. 

## 7. Marine-Derived Compounds

The awareness of the need to create beauty from inside to outside contributed to the emergence of such terms as nutricosmetics and cosmeceuticals [177]. Unique chemical compounds with superior biological properties have often been found in marine resources rather than terrestrial ones [177,178,179]. Fish oils are valuable sources of omega-3 fatty acids, while crustaceans and seaweeds supply the antioxidants such as carotenoids and phenolic compounds [180]. For instance, carotenoid astaxanthin obtained from crustaceans or other marine organisms possesses substantial antioxidant and anti-wrinkle effects [181]. It improves skin elasticity and reduces wrinkle formation due to immune-modulating, anti-inflammatory, and DNA repair effects [182]. Astaxanthin can also prevent neurodegenerative disorders [183,184]. Laminarin extracted from brown algae attenuates UV-induced skin damage [185]. 

Moreover, marine microorganisms such as microalgae, bacteria, and myxomycetes can be sources of antibacterial, antiviral, antitumoral, and antioxidant chemicals [180,186]. Thus, the extract from the green microalgae *Dunaliella salina* can prevent skin aging due to its anti-inflammatory and antiglycation properties [187]. The carbohydrates derived from marine algae benefit skin health [188].

## 8. Honeybee Products

Scientists have repeatedly pointed to the longevity-promoting properties of different honeybee products such as royal jelly, bee pollen, propolis, and honey [189,190,191]. In the frame of reviewing the use of bee products in dermatology, royal jelly has a multitude of pharmacological effects such as anti-inflammatory, antiallergenic, antibiotic, and antiaging. Collazo et al. reported the antioxidant, anti-lipidemic, antiproliferative, antimicrobial, anti-inflammatory, immunomodulatory, neuroprotective, antiaging, and estrogenic activities of royal jelly obtained from bees [192]. The abovementioned properties are mainly due to protein, carbohydrate, and lipids, but also macro- and microelements, vitamins, polyphenols, and terpenic volatiles in lower amounts. Kunugi and Mohammed, after researching a number of investigations from animal models to humans, found that the ingredients of royal jelly can promote longevity and healthy aging [193]. Royal jelly extended the lifespan of *Drosophila melanogaster* [189]. *In vivo* studies also showed estrogen-like effects, making it the basis for anti-menopause use [189].

## 9. Mushrooms

Zinc-containing polysaccharides from the edible mushroom Maitake (*Grifola frondose*) possess anti-aging abilities revealed *in vivo* [194]. The polysaccharides extracted from Maitake (*Grifola frondosa*) were approved for immunotherapy in the treatment of human cancer [195]. The complexes of polysaccharides extracted from such mushrooms as *Agaricus blazei* and *Ganoderma lucidum* also possessed anti-aging effects [196].

## 10. Probiotics 

*Enterococci*, *lactobacilli*, and *bifidobacteria* are the most commonly used probiotics that are the natural residents of the human organism [197,198]. Their possibility to modulate the skin and gut microbiota can prevent inflammation, allergic diseases and boost antiviral immunity [197,199,200]. It is important to highlight the effective role of microbiota composition in health status [201]. Host metabolism and insulin resistance might be affected by the gut microbiota through nutrient uptake and digestion and therefore be a causal factor in diabetes and obesity [202]. The microbiota also has been involved in helping to scavenge toxins, fight pathogens, and decrease inflammation, and it has a role in bowel diseases [200,203]. However, different studies suggest that the microbiota may influence aging, indicating that the health status could be improved by microbiota alterations with the help of probiotic bacteria reported in yogurt [204]. However, variations in the gut composition of microbiota have been reported between the elderly and young in several studies, indicating microbiota’s influence on human aging. However, most causal mechanisms are largely unknown for the correlative nature of probiotic bacteria and aging. 

## 11. Drinking Water

Drinking enough water as part of the diet also benefits health, especially in age, as dehydration is associated with higher disability in older people [205,206]. Chlorine levels in public drinking water may play a defining role in human health [207]. The regular addition of hypochlorite or chlorine to drinking water is applied extensively as the most effective means of delivering safe drinkable water due to its powerful anti-microbial functions. It could produce a continuing disinfectant that induces an endurable effect in the distribution system. According to toxicity studies, chlorine levels applied for metropolitan water treatment are safe for the individual. However, no studies are evaluating if chlorine levels from persistent exposure to tap water are also safe for colonized microorganisms in the gastrointestinal tract. It has been reported that gut dysbiosis could be induced because of constant exposure to low chlorine contents, affecting the microbiome, which has now been associated with different chronic non-communicable diseases [207]. Moreover, microbiome profiling of drinking water show the potential environmental source for the incidence of inflammatory bowel disease [208], indicating the important role of microbiological drinking water quality for human health [209].

## 12. Natural Substances Dangerous to the Human Body 

Common toxins such as tobacco, alcohol, air and water pollution, several medications, and contact with poisonous plants, animals, fungi, and microorganisms adversely affect human health, accelerating aging [210]. Natural toxins comprise substances toxic to humans, which appear in microbes, animals, lower and higher fungi, algae and plankton, and plants. The Joint FAO/WHO Expert Committee on Food Additives (JECFA) carries out risk assessments in food and sets the tolerable intake level for natural toxins [211].

Bacteria of the genus *Clostridium* produce the botulinum toxin, which binds glycoproteins on cholinergic nerve endings and blocks the production of acetylcholine. These neurotoxic effects gave rise to their application for treating ailments caused by unusual muscle contractions. Botulinum toxin type A has a wide application in cosmetology to improve the appearance of facial wrinkles, yet the toxin is dangerous if used in a non-standardized and approved way by professionals with full expertise [212].

Tetrodotoxin, known for its local anesthetic effect, is found in pufferfish (Fugu). Its systemic toxicity develops neural blockage and muscular weakness, leading to diaphragm paralysis [213]. Tetrodotoxin is a potent blocker of voltage-gated sodium channels. New medicines containing the toxin are under development, including its encapsulated dosage forms of microparticles and liposomes conjugated to gold nanorods [212].

Aquatic biotoxins include algal toxins, which can cause diarrhea, vomiting, paralysis, etc., and ciguatoxins, produced by dinoflagellates. Symptoms of ciguatera poisoning are nausea, vomiting, and neurologic signs [211].

Cyanobacterial toxins, presenting a high risk to humans, are the neurotoxic alkaloids (anatoxins and paralytic shellfish poisons), the cyclic peptide hepatotoxins (microcystins), and the cytotoxic alkaloids (cylindrospermopsins). Microcystins cause acute liver injury and are active tumor promoters; cylindrospermopsin is a potential carcinogen [214].

The neurotoxin saxitoxin and its derivatives, mainly neosaxitoxin, referred to as paralytic shellfish toxins, are found in prokaryotic cyanobacteria *Aphanizomenon flos-aquae* and eukaryotic dinoflagellates; these toxic substances can cause paralytic shellfish poisoning and saxitoxin pufferfish poisoning due to their ability to bind to the voltage-gated sodium channel [215,216].

The main toxins produced by filamentous cyanobacteria *Anabaena flos-aquae* are anatoxin-a and homoanatoxin-a, able to make water medium toxic for animals [217]. The purified toxin of the blue-green alga *Microcystis aeruginosa* (syn. *Anacystis cyanea*), administrated parenterally, developed extensive liver lobular hemorrhage and death in mice [218]. Filamentous cyanobacterium *Nostoc sp.* produced hepatotoxic peptides, which are types of microcystin-LR homologs similar to other cyanobacteria [219]. Data concerning the structure, sources, health impact, targets, and biological effects of marine neurotoxins are summarized in the review of Cusick and Sayler [216]. 

Mycotoxins, currently known as representing up to 400 structures, are produced by micro-fungi of the genera *Aspergillus, Fusarium,* and *Penicillium*, which can cause various diseases, including kidney and liver injury, congenital disabilities, cancers, and death in humans. Among the most potent mycotoxins are aflatoxins (AFB_1_, B_2_, G_1_, and G_2_), fumonisins (FB_1_, FB_2_, and FB_3_), ochratoxin A, trichothecenes, deoxynivalenol, zearalenone, patulin, citrinin, ergot alkaloids, and beauvericin [220,221,222,223,224]. Products of plant origin provide the natural substrates for fungi, which can be accompanied by mycotoxin development under suitable conditions [221,224,225].

Due to the content of muscimol and muscarin, within 6–24 h after ingestion of wild poisonous mushrooms, vomiting, diarrhea, visual disturbances, salivation, and hallucinations might develop; lethal sequelae are caused by potent toxicity of the mushroom toxins on the hepatocytes, renal cells, and neurons [211].

Diaz proposed a classification system for poisoning by highly toxic plants, dividing them into specific toxidromes: cardiotoxic; neurotoxic; cytotoxic; and gastrointestinal-hepatotoxic [226]. Among toxins of higher plants of phytotherapeutic importance are aconitine, strychnine, scopolamine, and anisodamine. The toxic herbs containing these alkaloids are currently applied after processing for pain reduction [227].

The various parts of *Datura stramonium*, especially its seed, are toxic due to the content of tropane alkaloids hyoscyamine, scopolamine, atropine, anisodamine, and iodine [227]. Alkaloid atropine, found in several *Solanaceae* plants, is a common cause of mid-latitude poisoning [228]. Edible solanaceous plants contain low levels of toxic glycoalkaloids solanine and chaconine [211].

Strychnine is the most toxic alkaloid of *Strychnos nux-vomica* seeds used as an analgesic and anesthetic remedy; 30–120 mg of strychnine is lethal for humans [227]. The median lethal dose of the toxic alkaloid is 1.5 mg/kg. Strychnine inhibits the postsynaptic receptors glycine, a main inhibitory neurotransmitter that can lead to severe muscle contractions, opisthotonic posturing, and respiratory muscle spasms [229]. Curare, a mixture of toxic alkaloids of *Chondrodendron* spp. or other *Menispermaceae* members and/or *Strychnos* spp., including strychnine, brucine, and tubocurarine, has been known as a muscle relaxant since it competes with acetylcholine for the binding site [212]. Pyrrolizidine alkaloids, which mostly occur in *Boraginaceae*, *Asteraceae*, and *Fabaceae* plants, can cause acute poisoning due to their ability to damage DNA and the formation of its adducts [230,231].

Various beans yield toxic lectins, which bind molecules to specific sugars. Ingesting a few raw red kidney beans can lead to severe vomiting and diarrhea [211]. Lectin ricin, one of the most potent phytotoxins in castor beans, inhibits intracellular protein synthesis. The LD50 of ricin toxin by the inhalation route is 3–5 µg/kg, while the oral one is 20 mg/kg [232,233]. 

Cyanogenic glycosides, another type of phytotoxins found in *Rosaceae* fruits, cassava, and sorghum, after ingestion can develop the signs of acute cyanide poisoning since hydrogen cyanide is formed as the result of enzymatic degradation of cyanoglycosides. A total of 0.5–3.5 mg/kg body weight of hydrogen cyanide is considered as an acute lethal dose for humans [234,235]. Linear furocoumarins, abundant in *Apiaceae* and *Rutaceae* plant species, are phototoxic agents that can cause sunburn and other acute reactions on sun-exposed human skin [236].

Although many natural ingredients benefit the body’s health, some are extremely strong or irritating and can cause certain health problems. Irritant contact dermatitis can be caused by mechanical means (trichomes, thorns, spines, sharp-edged leaves, etc.) or chemical agents (organic acids, calcium oxalate, protoanemonin, isothiocyanates, bromelain, essential oils, diterpene esters, alkaloids, naphthoquinone) from plants [237]. Substances derived from plants that may cause irritant contact dermatitis are present in significant content in many herbs: calcium oxalate in the leaves and flower stalks of daffodils, zambil, and cactus; isothiocyanates in horseradish, wasabi, papaya, garlic; essential oils of peppermint, lavender, etc.; lactone protoanemonine in buttercups; some alkaloids; organic acids such as citric (citrus), acetic (vinegar), formic, malic, salicylic acid, etc. Allergic contact dermatitis can be caused by allergenic components of chrysanthemum, daisy, dandelion, ambrosia, ivy, etc. [238,239,240]. Generally, the most important allergenic plant family is Asteraceae [238]. Severe dermatitis can also be provoked by photosensitivity as a result of the high reactivity of dermal tissues upon exposure to sunlight after ingestion or contact with UV-reactive plant secondary metabolites of heterocyclic or polyphenolic nature from *Ruta graveolens, Hypericum perforatum,* etc. [241]. 

Chronic exposure to trace elements can cause numerous adverse effects. Dietary exposure of about 300 µg/day of selenium may cause a hormone imbalance, neurotoxic, dermatological, and other side effects [242]. Data concerning the oncogenic characteristics of Se are contradictory [243].

Humic acid impedes the adhesion molecule expression by inhibiting NF-kappaB activation, which might partially contribute to immune and inflammatory disturbances in patients with the Blackfoot disease [244]. HA was demonstrated to cause echinocytic formation of human erythrocytes [245].

The α-Tocopherol β-Carotene and Retinol Cancer prevention (ATBC) study and the β -Carotene and Retinol Efficacy Trial (CARET) unexpectedly showed an increased risk of lung cancer and overall mortality in smokers who took supplements (20 mg) of β-carotene. The results of cyto- and genotoxic studies demonstrated that breakdown products of β-carotene are responsible for the development of carcinogenic effects [246,247,248]. Long-term internal use of *Aloe vera* can result in a laxative effect due to the content of anthraquinone glycosides [249]. Certain medications can interact positively or negatively with the glycosides contained in the drug. Rich purine alkaloid plant products demonstrate anti-aging and antioxidant potential [250]. Cardiac glycosides, despite their toxicity, exhibit the senolytic properties in experiments against age-related diseases [251]. Therefore, in administering potentially harmful plant constituents, it is very important to consider the famous Paracelsus’s quote, “Everything is a poison, nothing is a poison. It is the dose that makes the poison”. Improper dosage can provoke side effects in the human body.

## 13. Concluding Remarks

One of the main provoking factors for the body’s aging comprises the generation of highly reactive oxygen and nitrogen species resulting in oxidative stress due to the imbalance between pro- and antioxidants. Nevertheless, it is worth considering that the permanent intake of excessive dosages of isolated antioxidants can harm the organism. Therefore, it is of great importance to use scientific approaches in formulating a balanced diet, dietary supplements, and cosmetic procedures to maintain the antioxidant system of the human body. Unfortunately, the natural compounds which are the basis of maintaining the tone of youth are difficult to define and so neglected that they are worth remembering from time to time. So, using internally and/or externally the necessary amount of appropriate vitamins, minerals, amino acids, PUFA, probiotics, some phytoextracts, principles of aromatherapy, and enough volume of high-quality drinking water are crucial in counteracting the aging process. However, it should be noted that some natural components can be poisonous, allergenic, or irritating and cause certain health problems, especially in the case of overdose. 
